# Incidence of hemoglobinopathies and spatialization of newborns with sickle cell trait in Mato Grosso do Sul, Brazil

**DOI:** 10.31744/einstein_journal/2022AO6535

**Published:** 2022-05-06

**Authors:** Priscila de Matos Cândido-Bacani, Patrícia Medeiros Silva Grilo, Vanessa da Silveira Ramos, Michelly Zanchin, Indiara Correia Pereira, Josaine Sousa Palmieri Oliveira, Vitor Matheus Bacani, Edis Belini

**Affiliations:** 1 Universidade Federal de Mato Grosso do Sul Três Lagoas MS Brazil Universidade Federal de Mato Grosso do Sul, Três Lagoas, MS, Brazil.; 2 Instituto de Pesquisas, Ensino e Diagnósticos Associação de Pais e Amigos dos Excepcionais Campo Grande MS Brazil Instituto de Pesquisas, Ensino e Diagnósticos, Associação de Pais e Amigos dos Excepcionais, Campo Grande, MS, Brazil.

**Keywords:** Neonatal screening, Sickle cell trait, Spatial analysis, Infant, newborn, Residence characteristics

## Abstract

**Objective:**

To evaluate the incidence of variant hemoglobins of newborn samples from the Neonatal Screening Center in the state of Mato Grosso do Sul, Brazil, and to analyze the distribution and spatial autocorrelation of newborns with sickle cell trait.

**Methods:**

Samples from 35,858 newborns screened by the Neonatal Screening Center. The samples with inconclusive diagnosis were submitted to electrophoretic, chromatographic, cytological and molecular analyses. The spatial distribution analysis of newborns with sickle cell trait was performed by spatial autocorrelation.

**Results:**

A total of 919 newborns showed an abnormal hemoglobin profile; in that, ten genotypes had significant clinical impacts identified. Among the asymptomatic newborns, the sickle cell trait was the most frequent (incidence of 1.885 cases/100 newborns). The highest incidence rates were registered in the municipalities of Terenos, Figueirão, Corguinho and Selvíria. There was positive spatial autocorrelation between the proportion of declared individuals of black race/color and the incidence of newborns with sickle cell trait.

**Conclusion:**

The early diagnosis by neonatal screening and laboratory tests was very important to identify abnormal hemoglobin profiles and guide the spatial autocorrelation analysis of sickle cell trait newborns in Mato Grosso do Sul, serving as a support to anticipate health measures aimed to discuss efficient therapeutic behaviors and effective planning of municipalities with the greatest need for care, monitoring and orientations for affected families.

## INTRODUCTION

Sickle cell disease (SCD) is one of the most common severe monogenic conditions and affects millions of people worldwide.^(1,2)^ Due to its high prevalence, morbidity and mortality, the World Health Organization (WHO) has acknowledges SCD as a global public health problem. According to estimates, the number of newborns (NB) with SCD will reach 14.24 million, in 2050.^(3)^ In Brazil, SCD affects approximately 3,000 children annually.^(4)^

The term “sickle cell disease” is used to define a group of genetic abnormalities, which present hemoglobin S (Hb S) and clinical manifestations. These abnormalities include sickle cell anemia (SCA), determined by homozygous Hb S (Hb SS), which is the most common and severe clinical form of the disease; by double heterozygosity, characterized by the association of Hb S with other abnormal hemoglobin (Hb), such as D, C and E, and by interaction with thalassemia, for example Hb S/beta-thalassemia or Hb SS/alpha-thalassemia.^(5,6)^ On the other hand, heterozygosity for the sickle cell mutation, called sickle cell trait, refers to the inheritance of a beta S-globin gene and a normal beta-globin gene; it is asymptomatic and the most frequent among abnormal Hb profiles.^(6,7)^

The sickle cell results from a point mutation (A>T) in the beta-globin gene, in which the amino acid glutamic acid is replaced by a valine in the sixth position of the beta-globin chain, originating a Hb molecule with altered physical and biochemical features.^(1,7)^ The main clinical manifestations of SCA are due to chronic hemolytic anemia, vaso-occlusive phenomena, and acute splenic sequestration. Most clinical complications tend to appear after the first year of life, impacting on quality of life, morbidity and mortality^(4,6)^and, in Brazil, infections are identified as the most common causes of death in patients with SCA.^(8)^

In Brazil, the distribution of the S gene is quite heterogeneous and predominantly affects the black population,^(4,8,9)^with the highest prevalence in the Northern and Northeastern regions, and the lowest in the Southern and Southeastern regions.^(9)^ However, it is not exclusive to this population, due to the high degree of miscegenation of the Brazilian population.^(4,8)^

Neonatal screening, regulated by Ordinance 822/2001 of the Ministry of Health through the National Neonatal Screening Program (PNTN - *Programa Nacional de Triagem Neonatal*), is essential for the early diagnosis of abnormal hemoglobin profile in newborn (NB), and to enable the introduction of adequate prophylaxis and follow-up of symptomatic cases, to prevent severe complications and reduce SCD-related mortality.^(4,10)^ In this sense, the use of the Geographical Information System makes it possible to map the spatial distribution of Hb S through the application of spatial analysis techniques, and to contribute to planning of health actions and effective decision-making by health managers, since it enables visualizing the frequency of abnormal Hb in the state, leading to actions to prevent severe cases, focusing on regions with a high frequency of Hb S.

## OBJECTIVE

To evaluate the incidence of abnormal hemoglobin profiles of newborn samples from the Neonatal Screening Center of the Mato Grosso do Sul state, and analyze the distribution and spatial autocorrelation of newborns identified with sickle cell trait.

## METHODS

For this study, NB samples submitted to the *Instituto de Pesquisas, Ensino e Diagnóstico da Associação de Pais e Amigos dos Excepcionais de Campo Grande* (APAE) [Research, Teaching and Diagnosis Institute of the Association of Parents and Friends of the Disabled] (Neonatal Screening Center of the state of Mato Grosso do Sul), from January to December 2019, were analyzed, obtaining the number of abnormal hemoglobin profiles for each municipality in the state.

The detection of abnormal Hb S in neonatal screening was carried out using high performance liquid chromatography (HPLC) (Trinity Biotech - Bray, Ireland, Ultra2 - Kit Genesys).

To confirm the laboratory diagnosis, all NB samples were reviewed at the Genetics and Molecular Biology Laboratory of the *Universidade Federal do Mato Grosso do Sul* (UFMS), Três Lagoas *Campus*. The samples of NB with non-conclusive results for abnormal Hb were submitted to electrophoresis in alkaline pH and acidic pH, cytological analysis, HPLC (Trinity Biotech - Bray, Ireland. Ultra2 Kit Resolution), and molecular analysis for abnormal Hb S, using the polymerase chain reaction associated with restriction-fragment length polymorphism (PCR-RFLP), allele-specific polymerase chain reaction (AS-PCR), gap-polymerase chain reaction (GAP-PCR), and globin gene sequencing by Sanger’s method for rare abnormal Hb.

The percentage of newborns screened in the state of Mato Grosso do Sul was calculated using data from the Information System on Live Births (SINASC - *Sistema de Informações sobre Nascidos Vivos*) of the Information Technology Department of the Unified Health System (DATASUS - *Departamento de Informática do Sistema Único de Saúde*) of Mato Grosso do Sul.^(11)^ The incidence coefficient of NB with hemoglobinopathies was calculated using data from the number of new cases divided by the total population at risk.

**Figure 1:**
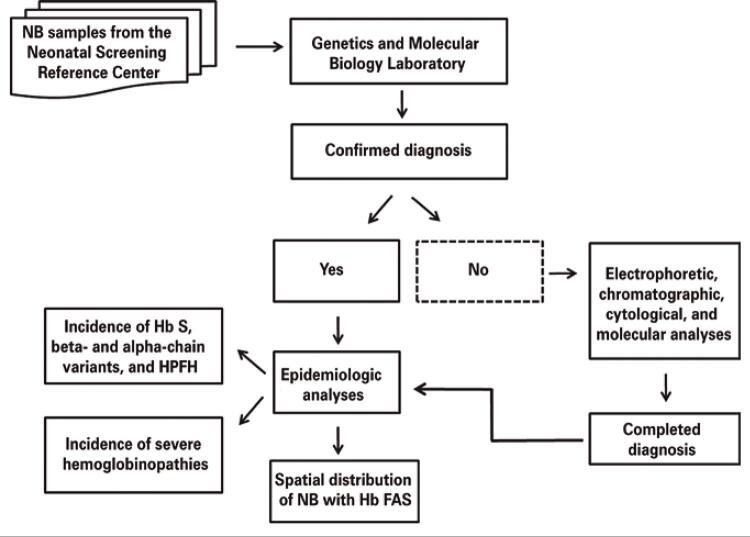
Methodologic flowchart

NB: newborn; Hb: hemoglobin; HPFH: hereditary persistence of fetal hemoglobin; Hb FAS: heterozygous for hemoglobin S.

To assess the occurrence of spatial patterns of NB with sickle cell trait in Mato Grosso do Sul, the Global Moran’s index (I) and the univariate and bivariate local indicator for spatial autocorrelation (LISA) through the spatial analysis per area (municipality) were applied. Statistical significance tests based on permutations were applied considering a significance level of p<0.05.^(12)^ Moran’s bivariate spatial autocorrelation analysis was used to assess possible spatial relations between the incidence of heterozygous hemoglobin profile for Hb S or sickle cell trait (Hb FAS) and the population declared as black or mixed race/color by the census of the Brazilian Institute of Geography and Statistics (IBGE)^(13)^ (Figure 1).

Statistical analyses of spatial autocorrelation were conducted using the GeoDa software, version 1.14.0.^(12)^The cartographic representation of the spatial distribution of incidence of NB with Hb FAS was prepared using the ArcGIS 10.2^(14)^software.

This is a cross-sectional, observational and descriptive study, approved by the Ethics Committee for Research on Human Beings of *Universidade Federal de Mato Grosso do Sul* (CAAE: 83109518.5.0000.0021, # 2.740,602). The parents or guardians signed the Informed Consent Form.

## RESULTS

The study included 35,858 NB submitted to the PNTN in the state of Mato Grosso do Sul, in 2019, covering 83.2% of live NB registered at SINASC/Mato Grosso do Sul. Of these, 919 NB (2.6%) presented abnormal hemoglobin profile (mean age: 12 days). A total of 693 NB (75.4%) were heterozygous for Hb S; in that, 676 Hb FAS (sickle cell trait; 73.6%), and 17 Hb FAS (1.8%) with coinheritance of alpha-thalassemia; nine (0.9%) had SCD (four with SCA – homozygous for Hb S – Hb FS and five were double-heterozygous for Hb S and Hb C – Hb SC). Other abnormal Hb profiles were present in 226 NB (24.6%) - in that, 211 NB (22.9%) with heterozygous Hb beta-chain variant (201 with Hb C, one Hb C and alpha-thalassemia coinheritance, four with Hb D-Los Angeles, two with Hb Korle-Bu, and two with Hb Osu-Christiansborg), one heterozygous for Hb Hasharon (alpha-chain Hb variant), and one NB with Hb FS profile. However, after molecular analyses, Hb S/HPFH type 2 (Ghana) was confirmed. And in relation to Hb C, one NB was homozygous for Hb C and four had Hb C/Beta + thalassemia (Table 1).

**Table 1:** Abnormal hemoglobin profiles identified in newborns from the state of Mato Grosso do Sul in 2019

Hemoglobin profile	n (%)	Incidence coefficient (%)
Hb FAS	676 (73.6)	1.885
Hb FAC	201 (21.9)	0.561
Hb FS	4 (0.4)	0.011
Hb FS (S/HPFH-2)	1 (0.1)	0.003
Hb FSC	5 (0.5)	0.014
Hb FC	1 (0.1)	0.003
Hb FCA (C/beta+ thalassemia)	4 (0.4)	0.011
Hb FAS+alpha thalassemia	17 (1.8)	0.047
Hb FA+Korle Bu	2 (0.3)	0.006
Hb FA+Osu-christiansborg	2 (0.3)	0.006
Hb FA+Hasharon	1 (0.1)	0.003
Hb FAC+alpha thalassemia	1 (0.1)	0.003
Hb FAD-Los Angeles	4 (0.4)	0.012
Total	919 (100)	

Table 1 depicts the incidence of 13 abnormal hemoglobin profiles identified in NB in the state of Mato Grosso do Sul, in 2019, the incidence of the Hb S allele being 1.957 cases/100 NB, and of SCD 0.025 cases/100 NB. The incidence of other abnormal Hb profiles in NB was 0.630 cases/100 NB.

**Figure 2:**
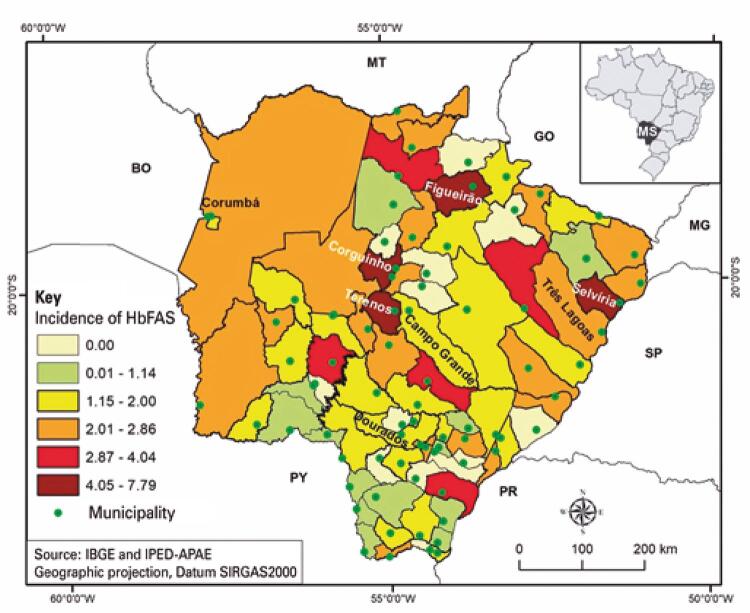
Incidence of newborns with sickle cell trait in municipalities of Mato Grosso do Sul, in 2019

Figure 2 presents the spatial distribution of the incidence of NB with Hb FAS in municipalities of Mato Grosso do Sul. Of the 79 municipalities of Mato Grosso do Sul, in 66 (83.5%), NB with sickle cell trait were identified in 2019. The highest rates were in Terenos, Figueirão, Corguinho and Selvíria. The rates ranged from 4.05 to 7.79 cases/100 NB. The lowest incidence rates (0.01 to 1.14 cases/100 NB) were found in the municipalities of Rio Verde de Mato Grosso, Inocência, Bela Vista, Antônio João, Jardim, Aral Moreira, Amambai, Coronel Sapucaia, Paranhos, Itaquirai, Eldorado, Glória de Dourados and Angélica.

Hb FAS: heterozygous for hemoglobin S.

**Figure 3:**
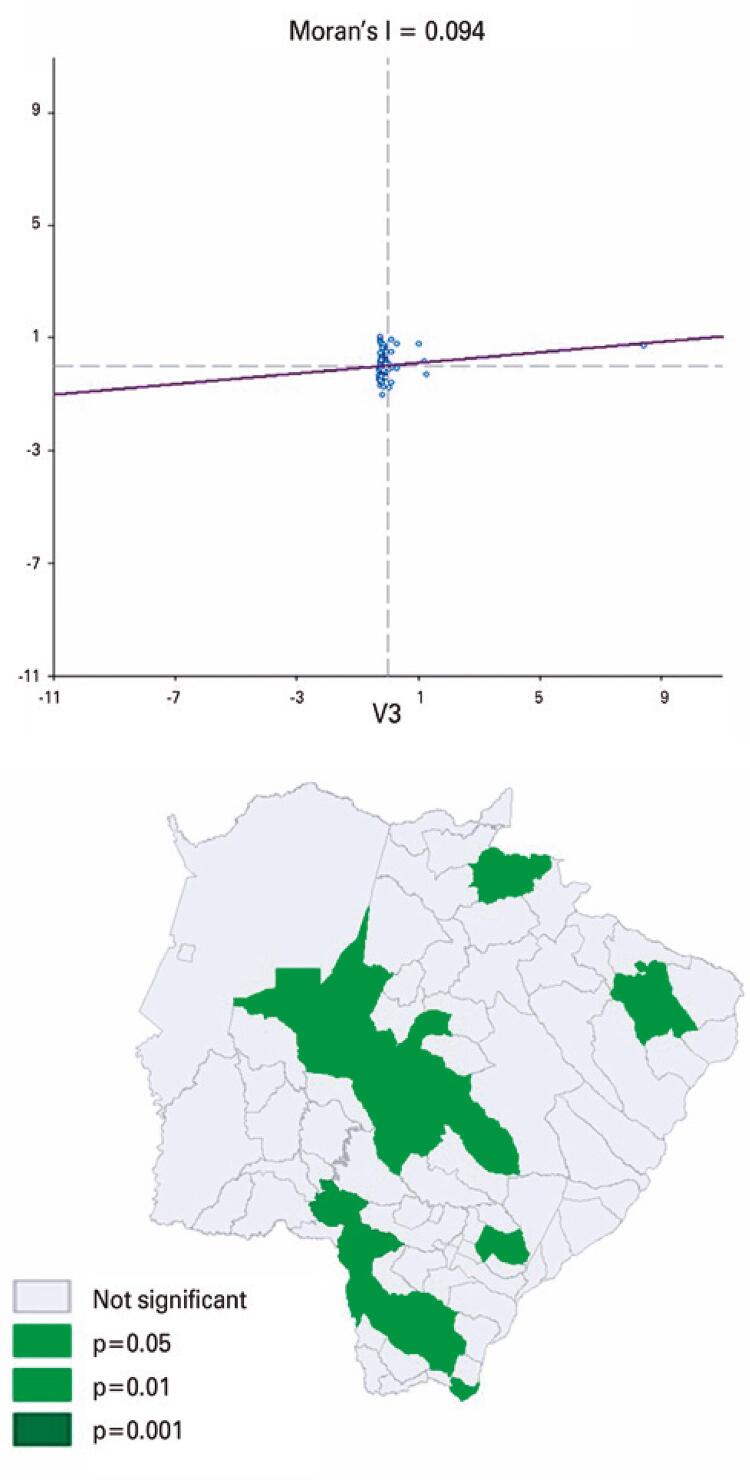
Dispersion chart and bivariate local Moran (LISA) significance map for the incidence of newborns heterozygous for hemoglobin S in Mato Grosso do Sul, in 2019, and population of subjects declared as black (race/color) (IBGE, 2010)(13)

Global Moran´s (I) and univariate local (Moran LISA) spatial autocorrelation were not significant, 0.079 and 0.080, respectively (p>0.05). However, the bivariate Moran LISA method between NB with Hb FAS genotypes and individuals declared to be black (race/color)^(13)^ indicated autocorrelation at the local level and spatial clusters (Moran index 0.094; p≤0.05) (Figure 3). The significance values of the local indices were classified by municipality as non-significant or significant (≤0.05). When considering subjects declared to be of black or mixed race/color, no significant spatial autocorrelation was found (Moran’s index 0.082; p>0.05).

**Figure 4:**
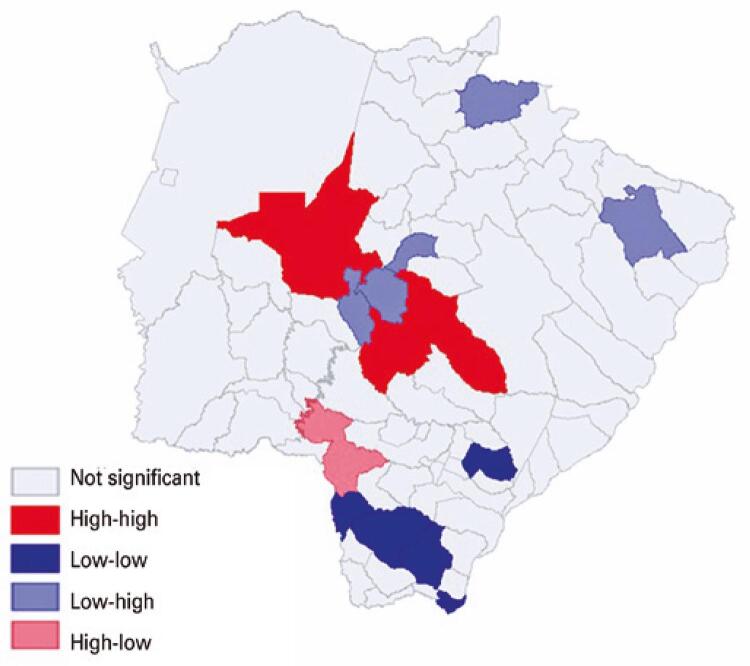
Map of bivariate local Moran autocorrelation (LISA) for the incidence of newborns heterozygous for hemoglobin S in Mato Grosso do Sul, in 2019, and population of subjects declared as black race/color (IBGE, 2010)(13)

The municipalities of Aquidauana, Campo Grande and Sidrolândia presented high-high autocorrelation, while Aral Moreira, Amambai, Iguatemi, Ivinhema and Mundo Novo had low-low autocorrelation. The municipality of Ponta Porã presented high-low autocorrelation, and Dois Irmãos do Buriti, Terenos, Rochedo, Alcinópolis and Inocência presented low-high autocorrelation. The other municipalities indicated in gray color were classified as having no significance, since they presented the same pattern of spatial significance (Figure 4).

## DISCUSSION

In this study, the incidence of abnormal hemoglobin profiles was determined in NB submitted to the test for hemoglobinopathies at the Neonatal Screening Center, in the state of Mato Grosso do Sul, in 2019. Of the 919 NB with abnormal hemoglobin profile, ten (1.09%) presented genotypes that will develop clinical complications; in that, nine NB with SCD (four with SCA – Hb FS and five were double-heterozygous Hb S and Hb C – Hb SC), and one NB homozygous for Hb C (Hb FC). Among the asymptomatic subjects, the Hb FAS genotype (sickle cell trait) was the most frequent, with 676 (73.6%) NB, an incidence of 1.885 cases/100 NB. The sickle cell trait was found in 66 municipalities of Mato Grosso do Sul, with the highest incidence rates registered in the municipalities of Terenos, Figueirão, Corguinho and Selvíria. The incidence of live births with hemoglobinopathies detected by the PNTN varied widely among Brazilian states, with Hb S being the most frequent of the Hb variants.^(15-18)^

The highest incidence rates of NB with SCD occur in states with a higher concentration of Afro-descendants, such as Bahia. In the state of Mato Grosso do Sul, the incidence of SCD and sickle cell trait was 1:8,300 and 1:70, respectively.^(18)^In our study, the proportion of live births with SCD was 0.025 case/100 NB, and the incidence of the S allele was 1.957 cases/100 NB.

In Mato Grosso do Sul, neonatal screening for SCD, carried out by the APAE Research, Teaching and Diagnosis Institute, began in 1997, that is, before the PNTN, established by the ministerial Ordinance 822 of June 6, 2001.^(16)^ The inclusion of hemoglobinopathies in the PNTN represents recognition of the relevance of this disease as a public health problem in Brazil.^(19)^

The PNTN coverage rate in Mato Grosso do Sul in this study was 83.19%, with an average coverage rate of 88.71% in the period 2001 to 2015.^(16)^In Brazil, the national average coverage in 2014 was 84% among live NB in the public health system.^(20)^ According to Eller et al.,^(21)^ in Brazil, the goal of 100% coverage of PNTN is still a challenge, and the same can be seen in more developed countries, such as Canada and Belgium.^(21)^It is worth emphasizing that, in this study, the samples analyzed were of NB seen at the public health system, the incidence of hemoglobinopathies in the private system being unknown.

Neonatal screening is key for early diagnosis of hemoglobinopathies in newborns before the onset of clinical symptoms, as well as the identification of asymptomatic heterozygous individuals who carry a beta S-globin gene, which can be transmitted to their offspring.^(4 ,10,20,22)^

Heterozygosity for the sickle cell mutation is considered a relatively common and clinically benign condition,^(16,23)^ and the geographic distribution of this “silent” population is essential for understanding the behavior of this gene in the population.^(24)^

This study was the first to assess the spatial distribution of NB with sickle cell trait in the state of Mato Grosso do Sul. Spatial analysis is an important tool for the study of genetic diseases, serving as a support to anticipate health measures and decrease negative impacts of hemoglobinopathies in the population.^(24)^

The global spatial autocorrelation of Hb FAS in the municipalities of Mato Grosso do Sul (Moran index; p value >0.05) was not significant. Therefore, there is no evidence, for the period analyzed, that the incidence of Hb FAS in a particular municipality is spatially correlated with the incidence of Hb FAS in neighboring municipalities. Although global indicators of spatial autocorrelation have one single value, when there is a large number of areas (all municipalities in the state), it is very likely that different spatial regimes will occur in sub-regions, which can be demonstrated by local indicators of spatial autocorrelation.^(25)^

The spatial distribution of NB with sickle cell trait presented local spatial autocorrelation with the proportion of individuals declared to be black (color/race). However, when considering the combined population declared black and mixed, there was no spatial autocorrelation. The existence of spatial patterns in NB with sickle cell trait was demonstrated by Leite et al.,^(24)^in a study carried out in the state of Sergipe. In that investigation, Hb FAS showed a positive spatial correlation with the percentage of self-identified non-white individuals.

High-high spatial regimes were found in the municipalities of Aquidauana, Campo Grande and Sidrolândia, that is, in these towns the high proportion of individuals declared to be black (race/color) showed a positive spatial correlation with the incidence of NB with sickle cell trait in Mato Grosso do Sul in the study period. The municipalities of Aral Moreira, Amambai, Iguatemi, Ivinhema and Mundo Novo indicated low-low autocorrelation. The frequency of Hb S found in this study reflects the ethnic diversity and the high degree of miscegenation that occurs in the Brazilian population.^(26)^

The municipalities of Aquidauana and Campo Grande have remnant settlements of *Quilombos*, the traditional peoples and communities of African origin.^(27)^ The lack of miscegenation in *Quilombola* communities may contribute to the maintenance of the high incidence of Hb S in these regions.^(24)^ The high positive spatial correlation (high-high) seen in Aquidauana and Campo Grande may be related to the presence of *Quilombola* communities in these municipalities.

Nonetheless, although the S gene predominantly affects the black population, it is not exclusive to that population, due to the different levels of racial mixing among Brazilians in each region of the country,^(4,8)^ what may explain the low-low autocorrelation patterns found in our study.

The state of Mato Grosso do Sul is located in the Mid-Western Region of Brazil, bordering the states of Mato Grosso, Goiás, Minas Gerais, São Paulo and Paraná, in addition to Bolivia and Paraguay.^(28)^ The population of the state typically is highly heterogeneous, as a result of demographic growth and increasing complexity of racial heterogeneity, marked by intense migratory flows in different historical contexts,^(29,30)^ what may explain the differences between Moran’s global and local indicators.

## CONCLUSION

The National Neonatal Screening Program of the State of Mato Grosso do Sul, associated with electrophoretic, chromatographic, cytological, and molecular analyses carried out at the Laboratory of Genetics and Molecular Biology of the *Universidade Federal do Mato Grosso do Sul*, Três Lagoas *Campus,* was extremely important for the characterization and early diagnosis of abnormal hemoglobin profiles in newborns in that location, which, in the neonatal period, would be clinically undetectable. In addition, from the analysis of the distribution and spatial autocorrelation of newborns with sickle cell trait in Mato Grosso do Sul, it is possible for managers to define effective public health actions, aimed especially to implement preventive practices and family education, contributing to reducing morbidity and mortality rates and to better quality of life of the population.
